# Accelerated Canine Retraction by Using Mini Implant With Low-Intensity Laser Therapy

**DOI:** 10.7759/cureus.33960

**Published:** 2023-01-19

**Authors:** Divya S Kharat, Sunil K Pulluri, Reshu Parmar, Deval M Choukhe, Salman Shaikh, Mayuri Jakkan

**Affiliations:** 1 Orthodontics, Dr. D. Y. Patil Medical College, Hospital & Research Centre, Pune, IND; 2 Orthodontics, Pandit Deendayal Upadhyay Dental College, Solapur, IND

**Keywords:** photo-biomodulation, low-intensity laser therapy, biomodulation, analgesics, accelerated orthodontics

## Abstract

Background

The continuous increase in demand for reduced treatment times has led researchers to think in terms of “accelerated orthodontics.” Generally, the duration for fixed orthodontic treatment is around two to three years. Prolonged use of braces leads to external root resorption, a high risk of caries, and decreased patient compliance. Therefore, finding an optimal supplementary approach to achieve faster tooth movement is still considered a subject of interest. Low-intensity laser therapy (LILT) is one of the non-invasive surgical techniques in the field of accelerated orthodontics. Low-level laser therapy (LLLT) has demonstrated faster healing, less bleeding, and biostimulation and anti-inflammatory effects. According to all studies, it accelerates tooth movement, thereby reducing braces treatment time. It is simple, safe, and minimally invasive. Despite these pieces of evidence, studies have shown variable findings in low-level laser therapy. This study evaluates the effect of LLLT on accelerated orthodontics in comparison with conventional canine retraction. An aluminum gallium arsenide-type diode laser with a wavelength of 940 nm has been used in this study.

Methodology

This study was conducted using the split-mouth method, which included 20 patients with permanent dentition who required first premolar extractions. A miniscrew implant was placed on both the right and left sides for maximum anchorage. Irradiation doses were applied on days 0, 3, 7, and 14 of the first month. Subsequently, irradiations were given every 15 days until the canine’s retraction was complete in the test group.

Results

The study results three months after the canine retraction in the test and control groups (M1) were 0.81 ± 0.03 mm/month and 0.74 ± 0.04 mm/month, respectively, indicating a significantly higher rate of canine retraction in the test group than in the control group (P < 0.0001). The average increase in the amount of tooth movement at three months was 40.1% and 36.3% in the test and control groups, respectively. However, the average increase in the amount of movement of teeth following canine retraction was 100% in the test group and 68.2% in the control group. There were significant variations in the pain score between Day 1 and Day 3 (P = 0.003) in the test group; however, there was no analytic variation in the pain score between Day 1 and Day 30 in the test group (P = 0.18). The pain score between Day 3 and Day 30 was significantly lower.

Conclusions

It was concluded that the rate of canine retraction increases when it is combined with LILT-assisted accelerated orthodontics in comparison to conventional canine retraction. Although LLLT does not provide immediate pain relief, it relieves the sensation of pain after 24-72 hours. LILT is an innovative, non-invasive technique that allows rapid orthodontic tooth movement. The rate of canine retraction increases when it is combined with LILT-assisted accelerated orthodontics in comparison to conventional canine retraction using mini-implants.

## Introduction

Usually, the time period for fixed orthodontic treatment is two to three years. The prolonged use of braces leads to internal root resorption, a high risk of caries, and decreased patient compliance. Therefore, finding an optimal supplementary approach to achieve faster tooth movement is still considered a subject of interest [1]. Forces from orthodontic appliances bend bones (bone bending theory), and bioelectrical potential development occurs. This can be achieved through approaches such as lasers, vibration, direct electric current, and so on [2].

Surgical methods have been developed to shorten orthodontic therapy, such as elevating flaps and performing corticotomy using surgical burs. Although traditional corticotomy accelerates tooth movement, it is forceful. As an alternative, flapless corticotomies (surgical therapies without flap elevation) may provide equivalent therapeutic effectiveness with less stress [3,4]. Recent research has aimed to reduce orthodontic treatment duration by 20-50% by topically injecting biomaterials, employing electrical currents, low-level laser therapy (LLLT), or flap-lifting operations such as traditional corticotomy. Many flapless alternative strategies have been offered to prevent the invasiveness of corticotomy operations and reduce pain and suffering while perhaps the regional acceleratory phenomenon (RAP) is ongoing. Reinforced scalpels and mallets (concision), MOP, piezopuncture, and piezocision are alternate options. Corticision is a potential flapless tooth-accelerating procedure because of its periodontal health, simplicity, availability, and cheaper cost [5]. Surgical damage to the alveolar bone induces the RAP, lessening the bone’s resistance to orthodontic stresses and speeding therapy. Conventional corticotomy techniques reduce orthodontic treatment time; however, they cause interdental bone loss, gingiva loss, periodontal abnormalities, and neck and face hematomas [6,7].

Due to their aggressiveness, surgical methods expedite tooth movements but are not frequently chosen by patients or dentists. These methods need complete mucoperiosteal flap elevations. They can cause post-surgical pain, swelling, crestal bone loss, bone necrosis, edema, and gingival recession. Thus, several minimally invasive methods have been proposed in the literature, such as corticision, piezocision, microosteo-perforations, and laser-assisted flapless corticotomy [8]. With the widespread usage of surgically assisted orthodontic acceleration, the analysis of patient-reported outcome measures has become very important to ensure patient acceptance and satisfaction before adopting any acceleration procedure [9]. Laser dentistry has become prevalent now. Since 1988, Er:YAG lasers have been used as dental lasers [10].

Low-intensity laser therapy (LILT) is a simple method that can be easily used in dental practice for different purposes, such as pain reduction, enhancement of wound healing, and inflammation alleviation [11]. LILT's stimulatory action has been shown in recent research to promote bone regeneration around the mid-palatal suture while promoting rapid palatal expansion and collagen synthesis. So far, only a few short-term animal studies have been conducted to assess the effect of LILT on orthodontic tooth movement [12]. LLLT has demonstrated faster healing, less bleeding, and bio-stimulation and anti-inflammatory effects. According to some studies, it accelerates tooth movement, thereby reducing braces treatment time. Although surgical acceleration methods have been extensively investigated, the examination of non-surgical methods also forms a wide body of research. Information and evidence regarding the superiority of one acceleration method over another remain ambiguous. However, LILT is simple, safe, and minimally invasive. Nonetheless, studies have shown variable findings for LLLT [13,14]. Therefore, it is necessary to assess the outcomes of LILT in terms of the amount of tooth movement using absolute anchorage.

The aim of the current study was to assess the outcomes of LILT on the rate of movement of teeth orthodontically and its analgesic effects.

## Materials and methods

The sample size was calculated using Minitab Version 17 (Minitab Inc., State College, PA, USA), and the intended test was a paired-samples t-test. The smallest difference requiring detection in canine movement velocity was assumed to be 0.25 mm/week. With an alpha level of 0.05, a power of 80%, and a standard deviation of 0.248 mm/week, the target sample was set at 18 patients. However, 20 patients were included in the study. Moreover, the total number of sites for observation was 40 in the age group of 18-35 years in the outpatient clinic of the Department of Orthodontics and Dentofacial Orthopaedics of our institute. The procedure was explained to the patient in detail, and informed consent was obtained. The study was approved by the institute's ethical committee with IRB no. Iec/2020/ortho/222. Patients with permanent dentition who needed first premolar extractions and had a healthy periodontium having no clinical attachment loss were considered eligible. On the other hand, the exclusion criteria included patients with any systemic diseases, skeletal crossbite, chewing or para-functional habits, occlusal disturbances, impacted canines, and breakage of maxillary canine brackets that have not been replaced within three to four days. For every patient, the right- and left-extracted quadrants were randomly categorized into two groups. The side on which laser therapy was administered was concealed from the participants.

**Figure 1:**
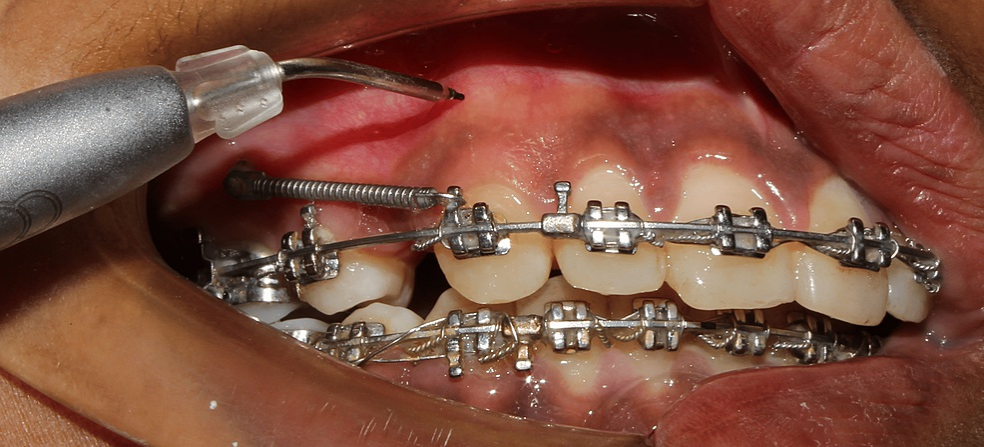
Low-level laser therapy application.

For Group I, the control side, no LILT was performed, whereas for Group II, the test side, LILT was performed. After banding the first molars, bracket bonding of both the maxillary arch and mandibular arch was done with MBT 0.022" x 0.028"-slot (Axim Brackets by Liberal Traders) prescription brackets. At regular intervals, patients were invited for follow-up, and archwires were replaced with a rectangular 0.019" x 0.025" stainless steel archwire. A mini-implant was placed on both the right and left sides for maximum anchorage (Denton, 7 mm in length and 1.3 mm in diameter). After 21 days of the stainless steel archwire placement, extraction spaces on both sides were measured using a digital vernier caliper. The distal surfaces of maxillary canines and mesial surfaces of maxillary second premolars were used as reference points. Retraction of individual canines was initiated using a nickel-titanium (NiTi) closed coil spring. Steel ligature wires were used to consolidate the incisors. Second premolars and molars were also consolidated to form a single unit. A sustained force of 150 g was measured using a Dontrix gauge, and a NiTi closed coil spring was inserted for canine retraction. Patients were told to call 911 if the spring broke or became dislodged [12]. LILT was initiated on the same day of the NiTi coil spring placement for its analgesic effect. A semiconductor-type diode laser such as aluminum gallium arsenide (EzlaseTM: A soft tissue diode laser, Biolase, USA) was used, emitting infrared radiation and operating according to the manufacturer’s recommendations with a wavelength of 940 nm. The laser wavelength should be set to 940 nm in continuous wave mode, with an output power of 100 mW and a 30-second exposure time for analgesic effects. For bio-stimulatory effects, the readings were kept at a wavelength of 940 nm, having an output power of 100 mW with a continuous wave and a 10-second exposure time (Figure 1).

Two irradiations were applied. At the baseline, the first irradiation was performed on the buccal side of the root portion of the canine on its middle third, while the second irradiation was applied on the middle one-third of the root of the canine along its palatal side. LILT was initiated on the third day to test the effects of bio-stimulation. A total of 10 irradiations were performed: five on the buccal side and five on the palatal side. For the alveolar process and periodontal fibers around the canines, the sites for laser irradiation were as follows: irradiation of the cervical one-third of the canine root (one dose on the mesial and one on the distal); irradiation of the apical one-third of the canine root (one dose on the mesial and one on the distal); irradiation of the middle one-third of the canine root (the center part of the root) once-a similar kind of irradiation was done on the palatal aspect-irradiation of the cervical one-third of the canine root (one dose on the mesial and one on the distal); irradiation of the apical one-third of the canine root (one dose on the mesial and one on the distal); and irradiation of the middle one-third of the canine root (the center part of the root) once. This approach was followed for all following visits. The laser therapy was performed on days 0, 3, 7, and 14 in the first month. Subsequently, laser irradiation was applied every 15 days till the retraction of the canine was complete on the test side [12]. To avoid operator differences in taking readings, all irradiations were administered by a single operator. The laser handpiece was kept on the control side without irradiation [12].

Two models were made for each patient: Model 1: before canine retraction; and Model 2: after canine retraction completion. The distances were recorded intra-orally at T0 (before canine retraction), T1 (three months following canine retraction), and T2 (upon canine retraction completion on the test side). The time interval between T0 and T1 is three months, during which the total movement of teeth was recorded. The rate of movement of teeth orthodontically was measured as the amount of tooth movement divided by the time taken. M1 is the rate of orthodontic tooth movement after three months, which was calculated as T0-T1 divided by three. M2 is the rate of movement of teeth orthodontically following the canine retraction on the test side, which was calculated as T1-T2 divided by the total number of months.

Each patient had a different response to the placement of the NiTi coil spring, and it was recorded using the visual analog scale for pain on days 1, 3, and 30 [12]. The research data were recorded and inspected using SPSS (Statistical Package For The Social Sciences) software v16.0. Intra-group variables were compared using paired t-tests. To compare the distances at T0, T1, and T2 in both test and control groups, a one-way analysis of variance (ANOVA; F statistics) was performed. Following these comparisons, posthoc multiple comparisons were conducted using the Bonferroni test.

## Results

Pain score comparisons between groups were performed using Wilcoxon signed-rank test. The significance was measured at 0.05 and 0.01 levels. Extraction spaces on both sides were recorded for both groups before the initiation of canine retraction, at three months of retraction, and after the completion of canine retraction in the test group (Table 1).

The mean distance between the mesial surface of the second premolar and the distal surface of the canine on the test side was 6.12 ± 0.41 mm (P < 0.0001) and on the control side was 6.14 ± 0.46 mm (P < 0.0001). The mean distance at three months of canine retraction on the test side was 3.68 ± 0.44 mm (P < 0.0001) and on the control side was 3.92 ± 0.48 mm (P < 0.0001). After complete canine retraction on the test side, the mean distance of extraction space remaining on the control side was 1.25 ± 0.26 mm (P < 0.0001) (Table 1). These values were noted in millimeters.

**Table 1:** Repeated measures ANOVA results for the mean distances (mm) between the mesial surface of the second premolar and the distal surface of the canine. HS: Highly significant; ANOVA: Analysis of variance

	Test	Control
T0	6.12 ± 0.41	6.14 ± 0.46
T1	3.68 ± 0.44	3.92 ± 0.48
T2	0.0 ± 0.0	1.25 ± 0.26
F-value	3078.4	3323.8
P-value	<0.0001	<0.0001
Inference	HS	HS

The amount of canine retraction (mm) at three months of canine retraction on the test side and the control side was 2.44 ± 0.096 mm and 2.22 ± 0.11 mm (P < 0.0001), respectively. which is highly significant (Table 2).

**Table 2:** Comparison of amounts of canine retraction (mm) between the test and control groups. HS: Highly significant; CI: Control interval

Variable	Test	Control	Mean Difference	95% CI of difference	t-value	P-value	Inference
T0-T1	2.44 ± 0.096	2.22 ± 0.11	0.22	0.15-0.28	6.68	<0.0001	HS
T1-T2	3.66 ± 0.44	2.64 ± 0.29	1.02	0.77-1.26	8.51	<0.0001	HS

The mean values of the rate of canine retraction after three months (M1) and that after its completion (M2) on the test side were calculated (Table 3). At three months after canine retraction in the test and control groups, M1 was 0.81 ± 0.03 mm/month and 0.74 ± 0.04 mm/month respectively, indicating a significantly higher rate of canine retraction in the test group compared to the control group (P < 0.0001). The average improvement in the amount of movement of the tooth in the third month was 40.1% and 36.3% in the test and control groups, respectively. On the other hand, the average improvement in the amount of movement of the tooth after canine retraction was 100% in the test group and 68.2% in the control group. Significant variations in pains score were noted between Day 1 and Day 3 (P = 0.003) in the test group. However, there was a significant variation in pain scores between Day 1 and Day 30 (P = 0.18) in the test group. On the third day of the procedure, there was a significant reduction in pain levels in the test group compared to the control group (P < 0.0001). On Day 30, significantly low pain levels were found in both groups (P = 0.003). However, the pain level on the test side was significantly less on Day 3 and Day 30 than on the control side.

**Table 3:** Comparison of the rates of canine retraction for the test and control groups. HS: Highly significant

Mean	Test	Control	t-value	P-value	Inference
M1	0.81 ± 0.03	0.74 ± 0.04	6.67	<0.0001	HS
M2	0.80 ± 0.03	0.58 ± 0.04	18.3	<0.0001	HS

## Discussion

Given that elderly patients are consistently seeking orthodontic treatment and the constant demand for reducing overall treatment duration, rapid orthodontic tooth movement is the primary focus of research scholars, as it has the advantage of fewer complications and greater patient compliance [13]. Mechanical or physical approaches include LLLT [15], direct electric current [16], and ultrasonic vibrations. A surgical approach is considered invasive and may lead to complications such as injuries to the surrounding vital structures, swelling, postoperative pain, infection, and patient non-compliance tendency [17]. The effects of LLLT are photochemical in nature and not thermal. A cell responds to LLLT by absorbing the laser light with a photo-acceptor molecule called a chromophore. The capacity of mitochondria is increased, leading to the generation of adenosine triphosphate (ATP). When the molecule absorbs light energy, it gets excited, and an accelerated rate of electron transfer occurs. Upregulated ATP levels result in more energy being available for metabolic processes in the cell. Coon et al. [17] and Kurol et al. [18] have demonstrated that both Cox-2 (Cyclooxygenase) and PGE-2 (Prostaglandin E2) increase RANKL (receptor activator of nuclear factor kappa beta) and decrease OPG (osteoprotegerin) levels. They also showed upregulated levels of Cox-2 in rodents following LLLT at the bone repair stage. Tooth movement associated with LLLT is shown to promote blood supply and regulate OPG/RANKL/RANK mechanisms. Chiari [19] used an 830 nm gallium-aluminum-arsenide diode laser to accelerate the rate of tooth movement. Further, Yamaguchi et al. [20], Fujita et al. [21], Youssef et al. [22], Yoshida et al. [23], and Doshi-Mehta et al. [12] showed that LILT accelerates tooth movement. However, LLLT had no additional benefits on orthodontic tooth movement, according to Limpanichkul et al. [24] and Heravi et al. [11]. The laser irradiation was done on days 0, 3, 7, and 14 of the first month. Similar to Doshi-Mehta et al. [12], in this study, laser irradiation was performed every 15 days till canine retraction was complete on the test side [12]. An absolute intervention timetable for LILT is not available; however, it has been stated that the application should be done at the initiation of orthodontic tooth movement [25]. Measurements were obtained intraorally from the distal surface of the maxillary canine to the mesial surface of the maxillary second premolars, which is cost-efficient, does not require any special equipment for measurement, and does not depend on any other factors such as bracket positioning, which was utilized as a reference for measuring extraction space by Cruz et al. [26]. According to Samuels et al. [27], NiTi closed springs provide more constant force than elastic modules, which is why it was used in this study. NiTi closed coil springs were also used by Genc et al. [28]. Immediate pain relief was not observed in this study. The treatment result was seen only after 24 to 48 hours. These observations supported the hypothesis that the effect of laser analgesia was significantly increased by the action of the laser on the inflammatory processes [29,30]. The pain score was significantly lower on the test side than on the control side on Day 3 and Day 30. Laser analgesia stabilized the nerve’s depolarizing capacity or its action on the biochemical and cellular effects of the inflammatory responses. LLLT can be used clinically to reduce treatment duration by increasing tooth movement. In addition, LILT can be used for analgesic effects for post-adjustment pain or after separator placement [30]. Further, LILT is non-invasive and requires no extra chairside time. This technique does not require any special assistance like in the case of surgical methods for accelerating tooth movement. LILT improves the amount of orthodontic movement of teeth in a physiological way, with no adverse effects on tooth vitality or surrounding structures. It has also been proven to be efficient from the perspectives of the patient and clinician.

Low-intensity laser irradiation requires a repeated irradiation schedule in order to be effective in tooth movement acceleration; however, it is difficult to sustain the patient follow-up schedule. For LILT, we require a low-intensity laser unit, which is not costly for the treatment in clinical practice. Good patient compliance is also required for the effectiveness of this technique in these long-term studies. As only 20 samples were included in this study, it is difficult to find a sample that meets all the specifications of the inclusion criteria. If possible, in future studies, the sample size should be increased to reduce errors. Due to poor compliance as a result of frequent visits, the laser irradiation intervention can be administered at three-week intervals such that there is no overlap with the regular follow-up visits. While measuring the distance of extraction spaces, newer methods such as CAD/CAM (computer-aided design/computer-aided manufacturing) can be used for more accuracy in measurement. Since MBT (McLaughlin-Bennet-Trevisi) mechanotherapy of 0.022" x 0.028" was used in this study, the self-ligation system can be used to further reduce treatment duration by minimizing friction during canine retraction. To check the orthodontic relapse tendency after LILT, more long-term studies can be conducted in the future.

Furthermore, the limitations of this study are that the nature of the canine movement (i.e., tipping, translation, or both) was not evaluated. Additionally, sex-related differences in canine retraction and patient-reported outcome measures were not considered, which future studies should focus on.

## Conclusions

By evaluating the efficiency of LILT and accelerated canine retraction in comparison with conventional canine retraction and on the basis of the study results, the following conclusions can be drawn. LILT is a non-invasive, innovative technique that allows rapid orthodontic tooth movement. The rate of canine retraction increases when it is combined with LILT-assisted accelerated orthodontics in comparison with conventional canine retraction using mini-implants. LLLT cannot provide immediate pain relief, but it shows pain relief after 24 to 48 hours.
